# Duration channels mediate human time perception

**DOI:** 10.1098/rspb.2011.1131

**Published:** 2011-08-10

**Authors:** James Heron, Craig Aaen-Stockdale, John Hotchkiss, Neil W. Roach, Paul V. McGraw, David Whitaker

**Affiliations:** 1 Bradford School of Optometry and Vision Science, University of Bradford, Bradford BD7 1DP, UK; 2 Visual Neuroscience Group, School of Psychology, University of Nottingham, Nottingham NG7 2RD, UK

**Keywords:** time perception, channels, visual, auditory, adaptation, duration

## Abstract

The task of deciding how long sensory events seem to last is one that the human nervous system appears to perform rapidly and, for sub-second intervals, seemingly without conscious effort. That these estimates can be performed within and between multiple sensory and motor domains suggest time perception forms one of the core, fundamental processes of our perception of the world around us. Given this significance, the current paucity in our understanding of how this process operates is surprising. One candidate mechanism for duration perception posits that duration may be mediated via a system of duration-selective ‘channels’, which are differentially activated depending on the match between afferent duration information and the channels' ‘preferred’ duration. However, this model awaits experimental validation. In the current study, we use the technique of sensory adaptation, and we present data that are well described by banks of duration channels that are limited in their bandwidth, sensory-specific, and appear to operate at a relatively early stage of visual and auditory sensory processing. Our results suggest that many of the computational principles the nervous system applies to coding visual spatial and auditory spectral information are common to its processing of temporal extent.

## 1. Introduction

There currently exists a marked dichotomy in our understanding of how we perceive the spatial and temporal properties of the world around us. For example, our knowledge of auditory location [1], visual position [2], size [3], orientation [4] and motion [5] processing have undergone step changes in recent decades. Relative to this body of spatial knowledge, our understanding of time perception is less well developed. This is perhaps surprising, given the critical importance of accurate temporal estimates for all aspects of sensory-motor processing, from speech perception to accurate guidance of our motor system. As recently highlighted [6], a likely cause for this dichotomy lies in the elusive nature of the neural mechanisms that underpin temporal processing. One profitable approach to probing these mechanisms is that of sensory adaptation [7]. For example, the application of adaptation techniques to questions surrounding spatial processing in the visual system have played a key role in revealing ‘channel’-based (CB) analysis and its underlying properties [8]. Equally, the auditory system appears to have dedicated channels for the estimation of location [9] and pitch [10,11]. A critical component of these CB systems is the presence of individual neural units that respond selectively to a relatively narrow range of afferent sensory information. For example, clusters of neurons in visual area V1 respond vigorously when presented with stimuli oriented close to vertical, but display relatively little activity when presented with horizontally oriented stimuli (i.e. their output is tuned) [12].

Returning to the temporal domain, it has been suggested that a similar approach could be used to construct sensory estimates of temporal extent. Specifically, a putative CB system for duration might contain neural units that respond selectively to a narrow range of stimulus durations centred on their preferred duration [13,14]. By comparing relative activation states across banks of these duration-tuned neurons, a ‘population response’ would emerge, which would signal the most likely perceived duration. Although behavioural evidence for human temporal judgements subserved by CB mechanisms remains sparse, it is noteworthy that several neurophysiological studies provide examples of visual [15,16] and auditory [17,18] neurons displaying bandpass duration tuning.

Such an arrangement would confer several advantages to the nervous system. First, population-based estimates tend to be relatively free of the potential ambiguity associated with absolute activity levels within individual channels (e.g. events with similar durations but differing levels of salience/intensity). Second, a system capable of extracting features of a population response is able to interpolate across individual channels, thus facilitating accurate estimates of duration over a range far greater than that predicted by its total number of constituent channels. However, while this framework appears theoretically feasible [13], it awaits experimental validation.

In the current study, we employ adaptation techniques to test predictions made by a CB model of temporal perception. Our findings show that recent sensory history plays a critical role in our perception of event duration. Adaptation to auditory or visual events of a consistent duration induces distortions in perceived duration that do not transfer to the non-adapted sensory modality. This effect is temporally tuned: when the relative durations of adapting and test stimuli are sufficiently different, the adapting stimulus fails to influence the perception of the test stimulus. Finally, we show that similar patterns of adaptation can be demonstrated across a range of durations spanning at least 160–640 ms, which form scaled, self-similar versions of one another. These findings provide strong support for CB models of time perception and display striking similarities to the features of the CB mechanisms known to mediate numerous perceptual estimates in the visual and auditory domains.

## 2. Materials and methods

### (a) Participants

Nine observers (four authors and five naive) participated in the main adaptation experiments while either three or four observers participated in subsequent control experiments (see figure legends for details).

### (b) Stimuli

The visual stimulus was a 100 per cent contrast isotropic Gaussian luminance blob (*σ* = 2.26° at a viewing distance of 57 cm) displayed against a uniform grey background (mean luminance: 47 cd m^−2^). The blob was presented at the centre of a gamma-corrected monitor screen (Sony Trinitron GDM FW900), which was driven by an Apple Mac Pro desktop computer running Mac OS 10.5. The visual stimulus was generated using Matlab 7.7 (Mathworks, USA) and Psychophysics Toolbox 3 (http://www.psychtoolbox.org). The auditory stimulus consisted of a burst of white noise presented via Sennheiser HD 280 headphones. Delivery of visual and auditory stimuli and the collection of observer's responses were controlled from within Matlab using custom software. The physical durations of visual and auditory stimuli were given rectangular onset–offset profiles. All timings were verified via simultaneous capture on a dual-channel oscilloscope.

### (c) Procedure

#### (i) Main adaptation experiments

**Figure 1.**
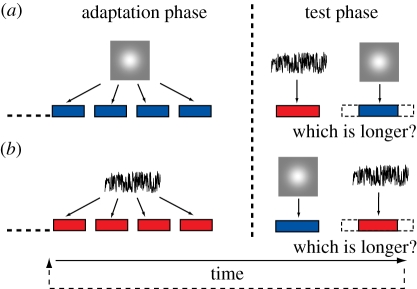
A schematic showing the paradigm used for the main adaptation experiments (figures 2–4; electronic supplementary material, figures S1, S2 and S5). Observers adapt to either (*a*) visual Gaussian blobs (in blue) or (*b*) bursts of auditory white noise (in red). The adaptation phase consists of 100 stimuli of identical duration (not shown) while the test phase consists of a reference stimulus from the opposing modality followed by a test stimulus (from the modality matching the adapting stimuli) of variable duration. In this example, adaptation stimuli are of a relatively short duration (e.g. 160 ms) relative to moderate duration reference (e.g. 320 ms). The last four adaptation stimuli are repeated between test phases to form a ‘top-up’ phase. For simplicity, the ISI is shown here as fixed, whereas in reality it varied randomly (see §2 for details).

**Figure 2.**
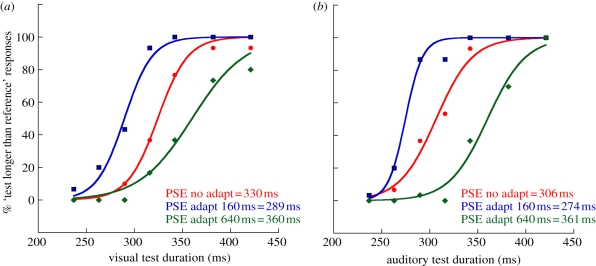
Sample psychometric functions from a single naive, representative observer (LEW) derived from duration discrimination judgements as to ‘which was longer, test or reference stimulus?’ (figure 1). These functions correspond to judgements made in the absence of adaptation (‘no adapt’, red data) or following adaptation to 160 or 640 ms (*a*) visual and (*b*) auditory duration stimuli (blue and green data, respectively). The effects of adaptation are quantified by differences in the point of subjective equality (PSE): the physical test duration corresponding to 50 per cent ‘test longer’ responses.

**Figure 3.**
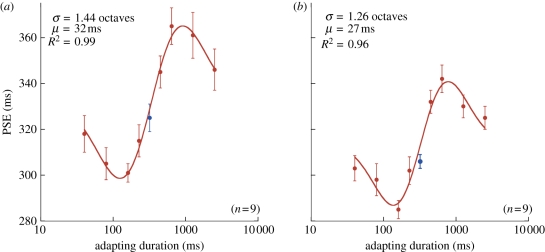
PSE data for a ‘no adapt’ condition (in blue) and following adaptation to (*a*) visual and (*b*) auditory stimuli with 40, 80, 160, 240, 400, 640, 1280 or 2560 ms durations (in red). Data are fitted with a curve based on the first derivative of a Gaussian (see §2 for details), which provides two important parameters: *μ*, the function's half amplitude (the magnitude by which the PSE deviates from baseline, or ‘after-effect magnitude’), and *σ*, standard deviation of the function (the temporal tuning of the adaptation). Error bars indicate the standard error of the mean.

**Figure 4.**
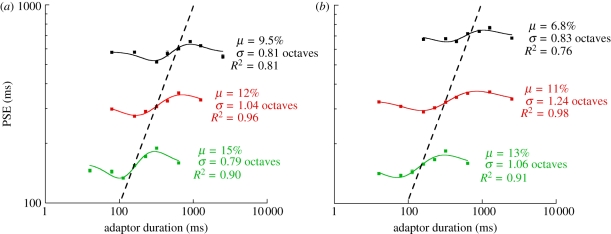
Tuning data for representative observer DW showing PSE values as a function of (*a*) auditory and (*b*) visual adapting duration for test duration ranges centred on 160 ms (green), 320 ms (red) and 640 ms (black). Note that red data points represent this observer's 320 ms test range data, which form part of the group average data shown in figure 3. The dashed black line represents a line of unit slope and illustrates the fact that the three curves can be superimposed on top of one another by sliding them along this line, indicating scaled self-similar mechanisms operating across test duration ranges. Error bars indicate the standard error of the mean.

Observers adapted to sequences of visual or auditory stimuli with a fixed duration before making two interval, forced choice duration discrimination judgements as to ‘which had the longer duration—test or reference stimulus?’ The test stimulus arose from the adapted sensory modality stimuli, whereas the reference stimulus arose from the non-adapted modality (figure 1). The duration of the reference stimulus remained fixed at 320 ms, while test stimulus duration varied in seven logarithmically spaced steps from 237 to 421 ms, which were randomly interleaved within a method of constant stimuli. Adapting duration was either 0 (‘no adapt’ baseline condition; figure 2, red data; figure 3, blue data), 40, 80, 160, 240, 400, 640, 1280 or 2560 ms and remained constant within each experimental block. Following an initial adaptation period comprising 100 adapting stimulus presentations, a 2000 ms pause signalled the start of the ‘top-up’ phase, which constituted the presentation of a further four adapting durations followed by reference and then test stimulus presentations. Receipt of the subject's duration discrimination judgement (via keyboard) triggered the presentation of the next top-up and test cycle. The inter-stimulus interval (ISI) between adapting, top-up, reference and test stimuli was randomly jittered in the range 500–1000 ms. Each block contained 10 repetitions of each test duration and three blocks were added together to give a total of 30 repetitions per condition. The presentation order of each block was selected by the presentation software in a pseudorandom order (figures 2–4; electronic supplementary material, figures S1, S2 and S5).

#### (ii) Control experiments

Figure 4 and electronic supplementary material, figure S2 comprise data from an experiment identical to that described above, with the exception that two further ranges of test durations were investigated. These ranges were centred on 160 and 640 ms, and were coupled with adaptation ranges spanning a three-octave range centred on the middle of the test duration range.

The reproduction experiment (electronic supplementary material, figures S3 and S4) was similar to the initial adaptation experiment, with two exceptions. First, the reference stimulus was removed such that the test stimulus now appeared immediately following the final top-up stimulus presentation. Second, the duration discrimination judgement was replaced with a reproduction task (see schematic shown in electronic supplementary material, figure S3) where observers depressed a keyboard button for a duration matching their estimate of the test duration. The effects of two adapting durations (160 ms, ‘adapt short’ and 640 ms, ‘adapt long’) on reproduction of the same seven test durations (237–421 ms—as per figure 2) were examined. This process was repeated for the four conditions shown in electronic supplementary material, figure S3.

The temporal frequency control experiment (electronic supplementary material, figure S5) involved adapting to a 160 ms duration visual stimulus and was similar to the main experiments (e.g. figure 2) with the exception that average ISI was increased from 750 ms (jittered between 500 and 1000 ms, as per the data shown in figures 2 and 3) to 1385 ms (jittered between 1135 and 1635 ms). This had the effect of reducing time-averaged stimulus presentation frequency from 1.1 to 0.72 Hz (as per the 640 ms data conditions shown in figures 2 and 3).

#### (iii) Data analysis

Psychometric functions comprising observer's duration discrimination judgement were plotted showing the proportion of ‘test longer than reference’ responses as a function of test duration (e.g. figure 2). These functions were fitted with a logistic of the form
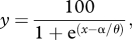
where *α* is the test duration value corresponding to the point of subjective equality (PSE; the 50% response level on the psychometric function) and *θ* provides an estimate of duration discrimination threshold (approximately half the offset between the 27% and 73% response levels). In this way, PSE values were obtained for all observers (figures 2–4; electronic supplementary material, figures S1, S2 and S5).

PSE data shown in figures 3 and 4 and electronic supplementary material, figure S2 were fitted with a curve based on the first derivative of a Gaussian, namely
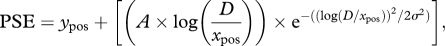
where *D* is the adaption duration, *σ* the standard deviation of the Gaussian, *A* a constant related to the amplitude of the function and (*x*_pos_, *y*_pos_) the origin of the function (note that when *D* = *x*_pos_, PSE = *y*_pos_). The maxima and minima of this function occur at adaptor durations ±*σ* log units from the origin (i.e. log(*D*/*x*_pos_) = ±*σ*). The half-amplitude of this function (*μ*), which represents the magnitude by which the PSE deviates from baseline (i.e. the size of any illusion), is therefore given by
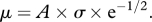


For the final reproduction experiment (electronic supplementary material, figures S3 and S4), the reproduced durations for each observer were averaged across test durations to give a mean reproduced duration (MRD) for each of the four conditions shown in electronic supplementary material, figure S3: (i) adapt visual duration, reproduce auditory duration; (ii) adapt auditory duration, reproduce visual duration; (iii) adapt visual duration, reproduce visual duration; and (iv) adapt auditory duration, reproduce auditory duration. For each of these conditions, the arithmetic difference between ‘adapt short’ and ‘adapt long’ was computed, then averaged across observers (*n* = 4), and forms the bars shown in electronic supplementary material, figure S4.

## 3. Results

### (a) Experience dependent duration plasticity

Psychometric functions for a representative naive observer are shown in figure 2. The lateral separation in opposite directions from the ‘no adapt’ baseline condition (figure 2, red data) of the green and blue functions shows that adaptation clearly modulates the proportion of ‘test longer’ responses in a repulsive fashion. Specifically, adapting to relatively short visual or auditory durations (160 ms; figure 2, blue data) induces an expansion in the perceived test durations (237–421 ms) that is subsequently viewed (figure 2*a*) or heard (figure 2*b*). The magnitude of this effect is reflected in the physical test duration corresponding to perceived equivalence between test and reference durations (the PSE). For example, after adapting to 160 ms durations, visual PSE shifts from 330 to 289 ms, while auditory PSE shifts from 306 to 274 ms. A reciprocal pattern is observed following adaptation to relatively long durations (640 ms), where test durations undergo perceptual compression (figure 2, green data). Average PSE shifts show this effect to be consistent across observers (*n* = 9; see electronic supplementary material, figure S1).

This pattern of repulsion-type after-effects is broadly similar to that observed following adaptation to consistent spatial information [8]. For example, prolonged viewing of visual stimuli of a relatively high spatial frequency induces a decrease in the perceived spatial frequency of subsequently viewed stimuli [3,19]. This parallel suggests that a CB framework may be consistent with the duration after-effects shown in figure 2. However, a further prediction of CB models concerns the relationship between after-effect magnitude and the degree of similarity between adaptation and test stimuli. This is exemplified by the finding that the influence of adaptation to consistent motion [20], orientation [21] and spatial frequency [22] is constrained to situations where adapt and test stimuli fall within a limited perceptual distance of one another. This distance is typically linked to the degree of selectivity associated with the system's individual component channels (i.e. their bandwidth). In many cases, these psychophysical measurements map closely onto the underlying response properties of neurons at multiple scales of the visual system [7].

### (b) Tuned duration after-effects

We investigated the possibility of duration-tuned mechanisms in humans by systematically altering the duration of the adapting stimuli while keeping the range of test stimuli constant. Average PSE values (*n* = 9) were extracted from the psychometric functions corresponding to each visual (figure 3*a*) and auditory (figure 3*b*) adaptation duration. Relative to the central ‘no adapt’ data point (in blue), increasing or decreasing the duration of the adapting stimuli induces a decrease or increase in PSE (in red), respectively. This reflects a relative contraction and expansion of perceived duration, which appears to increase in an approximately linear fashion over a limited range of adapter durations. Beyond this range, adaptation magnitude declines such that the longest and shortest adapters (40 and 2560 ms) induce changes in perceived duration approaching those observed in the no-adapt condition. This pattern of results is markedly similar across visual and auditory domains (cf. figure 3*a*,*b*).

In order to characterize these effects, a curve based on the first derivative of a Gaussian (see §2 for details) was fitted to the data that allowed extraction of several important parameters. While the amplitude of the visual and auditory functions—reflecting the magnitude of the adaptation effect—is similar, the bandwidths of the functions (in octaves) are slightly broader for vision than audition (1.44 versus 1.26). In other words, both modalities appear to possess approximately equivalent degrees of flexibility in response to duration adaptation, yet vision shows a greater tolerance to discrepancies between the duration of test and adaptor. Consistent with earlier reports [23–26], auditory durations are perceived as longer than their (physically identical) visual counterparts, irrespective of adaptation. This is reflected in the vertical offset between the two datasets, with a higher PSE indicating relatively shorter perceived duration.

### (c) Scaled, self-similar duration channels

In addition to the tuning features described above, channel- or filter-based perceptual systems are further characterized by a trend towards banks of overlapping channels that form self-similar, scaled versions of one another. For example, the bandwidth of channels responsible for processing auditory pitch [27,28] or visual spatial frequency [22,29,30] typically form a fixed proportion of the frequency to which channel is maximally responsive. When expressed in logarithmic terms, this gives rise to tuning functions that are approximately equivalent in appearance across a large range of stimulus parameters. Given that our range of test durations (237–421 ms) contains substantial overlap with biologically significant durations such as those thought to be critical for speech perception [31,32], effects shown in figures 2 and 3 may reflect duration mechanisms that are peculiar to this test range. Alternatively, if duration channels form a generalized feature of temporal judgements in the ‘automatic’ range [33,34], comparable versions of tuning data from figure 3 should be elicited by testing at different sub-second ranges. We tested this hypothesis by examining the effect of duration adaptation on two further ranges of test durations centred on 160 and 640 ms. Each test range was paired with a corresponding range of adapting durations that formed octave steps either side of the centre of the test range (see §2 for details). Results for one representative observer are shown in figure 4. For both modalities, longer (640 ms, black curve) and shorter (160 ms, green curve) test range data show a marked degree of similarity to the 320 ms range data (in red, as per figure 3). Specifically, despite small variations in bandwidth and amplitude values across the different test ranges, a remarkable degree of similarity is evident between the three functions. This pattern of results is replicated across observers as shown in electronic supplementary material, figure S2. Channels characterized by scaled, self-similar bandwidths are entirely consistent with the data shown in figure 4.

### (d) Sensory specificity

These adaptation effects appear to be limited to the adapting modality: if our duration distortions transferred between test and reference stimuli (i.e. from audition to vision and *vice versa*; figure 1) then both the test and (opposite sense) reference would be affected equally, resulting in no measurable effect. Nevertheless, it remains possible that a partial transfer across the modalities could mask larger after-effect magnitudes than those seen in figures 2–4. We investigated this possibility by replacing our duration discrimination judgements with a reproduction task (see schematic shown in electronic supplementary material, figure S3) where our reference stimuli were omitted. Instead, observers depressed a button for a duration that matched their estimate of the test stimulus's perceived duration (see §2 for details). Although reproduction tasks are associated with issues surrounding their criterion-dependent nature [35], by removing the relative nature of the intersensory comparisons made in figures 2–4, a more absolute measure of perceived time is made available. The results of this experiment are shown in electronic supplementary material, figure S4, where the effects of adaptation are expressed as the difference between MRDs following adaptation to 160 and 640 ms durations, with positive values indicating repulsive duration after-effects of the type observed in earlier figures and values close to zero indicating little or no effect of adaptation. While the reproduction data show some differences in the absolute value of the adaptation effects (cf. those observed with duration discrimination judgements), two key features of the data warrant consideration. First, the positive values observed for the within-modality, ‘intramodal’ adaptation conditions show that repulsive duration aftereffects are not peculiar to the methodology employed in earlier experiments. Second, these repulsive after-effects were only elicited when adaptation and test stimuli arose from the same sensory modality.

### (e) Duration adaptation or temporal frequency adaptation?

Recent evidence suggests that the perception of moderately paced rhythmic auditory patterns can be slowed down or speeded up via prior exposure to relatively fast or slow tone sequences [14]. Although our observers adapted to filled durations rather than rhythmic sequences, the combination of stimulus duration and an average ISI of 750 ms (jittered between 500 and 1000 ms) provides observers with an average temporal frequency (TF) that will vary with the duration of the adapting stimulus. For example, adapting to 160 ms stimuli introduces an average TF of 1.1 Hz, whereas a 640 ms adapting stimulus provides an average TF of 0.72 Hz. In order to ascertain whether TF after-effects contribute to the effects presented thus far, we designed a control experiment where visual adapting duration was fixed at 160 ms but average ISI was manipulated to provide a TF of 1.1 Hz (see §2 for details). If our adaptation effects are driven by a TF-based mechanism, we would expect to see equivalence between the 1.1 Hz (160 ms duration stimuli) condition and the 1.1 Hz (640 ms duration stimuli) condition. However, if our effects reflect genuine duration adaptation, the 1.1 Hz (160 ms) should share similarity with the 160 ms data shown in figures 2–4. The results are shown in electronic supplementary material, figure S5, where adaptation-induced shifts in PSE are plotted—relative to the 320 ms baseline condition—for the two different TFs and adapting durations. Clearly, the closest match in after-effect magnitude and polarity is between the 0.72 Hz (160 ms) and 1.1 Hz (160 ms) conditions. This finding confirms the underlying importance of event duration—rather than interevent TF—in generating the after-effects presented here.

### (f) Modelling the effects of duration adaptation

Adaptation-induced biases in perception are typically explained using a common set of assumptions: (i) stimulus properties are encoded by populations of neurons with distinct (though typically overlapping) tuning curves; (ii) adaptation selectively changes the responses of these neurons; and (iii) downstream mechanisms that decode (‘read out’) the activity of the population are unaware of these changes (for recent reviews see [36,37]). To determine whether it is possible to account for the effects of duration adaptation in a similar manner, we constructed a simple population coding model comprising sets of dedicated, modality-specific time channels. Our intention was to establish a model capable of quantitatively describing our psychophysical data with the smallest set of assumptions possible.

**Figure 5.**
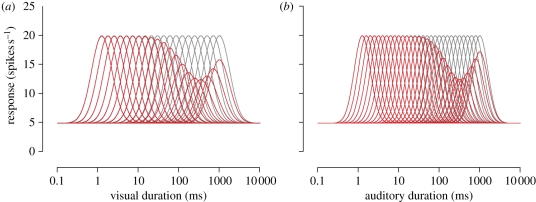
Model tuning curves for (*a*) visual and (*b*) auditory duration channels, with (red) and without (black) adaptation to a fixed duration.

We began by generating a population of neurons with log-Gaussian duration tuning for each sensory modality (figure 5). Physiological evidence has previously been reported for this form of duration tuning across a range of neural structures [15–18,38–40]. Preferred durations were arbitrarily set to range from 1 to 1000 ms in equal log steps. In different simulations, we varied the number of neurons (*n*) and the standard deviation (*σ*) of the tuning functions (fixed for each modality). Adaptation was modelled as a selective modality-specific reduction in response gain that was maximal at adapted duration (*A*_max_) and fell off with log-Gaussian profile (width set by *A*_*σ*_).

**Figure 6.**
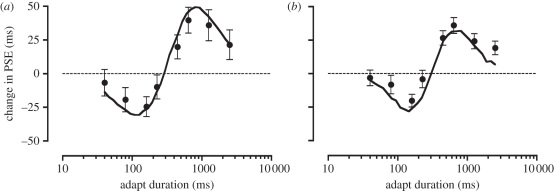
Comparison of experimental and model data. Data points show mean shifts in the PSE as a function of adaptor duration (re-plotted from figure 3). Solid lines show the predictions of the best-fitting model. (*a*) Visual: *n* = 20; *σ* = 0.25 (log units); *A*_max_ = 50%; *A*_*σ*_ = 0.45 (log units). (*b*) Auditory: *n* = 30; *A*_*σ*_ = 0.2 (log units); *A*_max_ = 50%; *A*_*σ*_ = 0.35 (log units).

Simulations mirrored the trial-by-trial structure of the psychophysical experiment, with a variable test stimulus presented to the adapted modality and a fixed 320 ms reference stimulus presented to the other modality. Neuronal responses were sampled from independent Poisson distributions centred on the value of each tuning curve for a given stimulus. We used a maximum-likelihood decoder [41] to generate a binary response on each trial. Figure 6 shows shifts in the PSE produced by the best-fitting model, alongside the corresponding empirical data. Clearly, the model is able to reproduce the repulsive shifts in perceived duration caused by adaptation and provide a reasonable approximation of the tuning of this effect (*R*^2^ = 0.9).

## 4. Discussion

In the current study, we present evidence that human estimates of visual and auditory temporal extent are mediated by a series of bandwidth-limited duration channels. Specifically, adaptation to fixed auditory or visual duration induces sensory-specific distortions of subsequently heard or viewed durations. The temporal spread of these distortions is limited by the temporal proximity of adaptation and test stimuli, a feature that underscores one of the key similarities between our duration-based effects and the classic literature characterizing CB visual [8] and auditory [42] processing. The fact that our data are well predicted by a generic CB model—without recourse to any novel features specific to temporal perception—emphasizes the similarities between established forms of CB perception and the effects presented in the current study.

### (a) Psychophysical context

A significant aspect of our data is the seeming ability of recent experience to selectively initiate both expansion and contraction of perceived duration. This bidirectionality differentiates our effects from other recent duration-based phenomena where sensory history also appears to play a role. For example, perceived duration can be manipulated via prior exposure to dynamic visual stimuli such as flickering patches [43,44] or drifting gratings [43–45]. Similarly, it has recently been argued that perceived visual duration depends on the extent to which a stimulus is deemed to be repetitive (i.e. its relative novelty) [46]. In both instances, experimental manipulations induce a unidirectional contraction of perceived duration but, as yet, have not shown reciprocal effects.

Our CB framework provides an explanation for earlier reports showing that repeated stimulation [47] or perceptual anchoring [23] can influence subsequent duration judgements. In addition, emerging evidence from perceptual learning experiments suggests that training-related increases in duration discrimination sensitivity are tied to durations close to the centre of the trained duration range [48]. Consistent with the data shown in figure 4 and electronic supplementary material, figure S2, the magnitude and bandwidth of these learning effects are approximately constant when expressed relative to the trained range (3–4% and 8–11%, respectively [48]). Similarly, one of the defining characteristics of duration judgements is the proportional relationship between duration discrimination thresholds and mean estimated duration (Weber's law for duration). Both of these effects show a degree of proportionality consistent with the data shown in figure 4 and electronic supplementary material, figure S2, and sit comfortably within a CB framework. Specifically, because channel bandwidth appears to vary in proportion to preferred duration, a system using these channels should show precisely the kind of Weber's law behaviour that is so often observed throughout the duration perception literature [49–52]. Interestingly, the amplitude of our effects shows a small but consistent tendency to decline with increases in test duration range (figure 4; electronic supplementary material, figure S2). On first inspection, this effect is perhaps suggestive of smaller levels of response gain reduction (figure 5) at longer test duration ranges. However, it is perhaps more likely to reflect an artefactual feature introduced by the increases in the total elapsed time between successive test stimulus presentations: longer test durations are paired with proportionally longer adaptation stimuli, which have the unintended consequence of lengthening test/re-test interval (figure 1). As such, it is reasonable to speculate that some degree of temporal decay is operating at the longer test duration ranges (cf. green, red and black data in figure 4 and electronic supplementary material, figure S2). In a further control experiment, we found that adaptation failed to influence perceived duration when our train of adapting stimuli was replaced with a single adapting stimulus. This finding appears to distance our effects from rapid, attention-dependent adaptation phenomena for which neural loci are thought to reside in extra-striate areas of the cortex [53–56].

### (b) Neural basis

To model our results, we have implemented a population coding framework in which stimulus duration is represented by the pattern of activation across a number of bandpass-tuned channels. A critical property of this framework is that stimuli of a particular duration stimulate (and therefore adapt) channels in a selective manner, allowing us to account for both the bidirectional (i.e. compressive and expansive) and tuned characteristics of the observed after-effects. While bandpass tuning of responses as a function of certain stimulus attributes is relatively common in sensory neurons, realizing this form of selectivity in the time domain poses unique challenges. Consider a collection of channels that each selectively responds once a particular time interval has elapsed following stimulus onset. Because of the unidirectional flow of time, the presentation of a stimulus will elicit a ‘domino effect’ in which channels respond successively one after another. In principle, repeated presentation of brief adapting stimuli might selectively adapt channels tuned to brief intervals, providing a basis for explaining expansions of perceived duration. However, as these same channels would also respond to each presentation of a longer adapting stimulus, achieving the selective adaptation required to produce compressions of perceived duration is problematic.

A simple mechanism that avoids this problem is a form of coincidence detection, in which channel activity is driven by simultaneous occurrence of sub-threshold excitatory events linked to stimulus onset and offset [18,38]. Within this scheme, different duration preferences can be generated by varying the latency of the onset event. Neurophysiological evidence for this type of tuning has been documented in the auditory midbrain (inferior colliculus) [17,18,57], primary auditory cortex [39,40], primary visual cortex [15] and prefrontal cortex [16]. Neurons within these areas display a phasic burst of spiking activity at stimulus offset, the magnitude of which is tied to the time elapsed since stimulus onset. This provides a direct physiological substrate for the modality-specific bandpass duration channels detailed in our model.

A variety of interval-coding mechanisms has been proposed, not all of which rely on dedicated timing channels [6,58]. Recent years have seen the emergence of distributed timing models, referred to as population clocks, which rely on time-dependent changes in the state of neural networks [33,59]. This approach offers considerable flexibility, permitting the continuous coding of elapsed time as well as an ability to discriminate between more complex temporal patterns. Because timing is represented in the dynamics of the entire network, it is not immediately obvious how our finding of selective duration after-effects could be accommodated within this framework. In some implementations of population clock models, different network states are read by output neurons that receive inputs from all the neurons in the network [60]. Feasibly, these output neurons could provide the basis of adaptable duration channels. However, neurophysiological evidence for this process is limited and it remains to be seen whether such a scheme could produce duration selectivity that overcomes the cascading activation problem discussed earlier.

One of the key advantages of a CB system is that the overlapping nature of these channels (figure 5) negates the need for the system to accommodate a large (potentially infinite) number of channels corresponding to every conceivable duration: by comparing differential activation levels across channels and extracting the population response [61,62], the system can interpolate between neighbouring channels' preferred durations. As outlined earlier, in addition to offering metabolic savings, such a system also affords high-resolution, low-ambiguity estimates of duration. However, this efficiency comes at a cost to the nervous system: sustained activity within individual channels (figure 5) induces repulsive biases in the population response to subsequently presented durations.

### (c) Conclusions

By using sensory adaptation, we have revealed a pattern of temporal perception that is indicative of a perceptual system underpinned by a range of overlapping duration-sensitive channels. We suggest that when formulating estimates of temporal extent, the human nervous system applies some of the same computational principles that are used in the processing of many of the fundamental—yet non-temporal—properties of the world around us.
